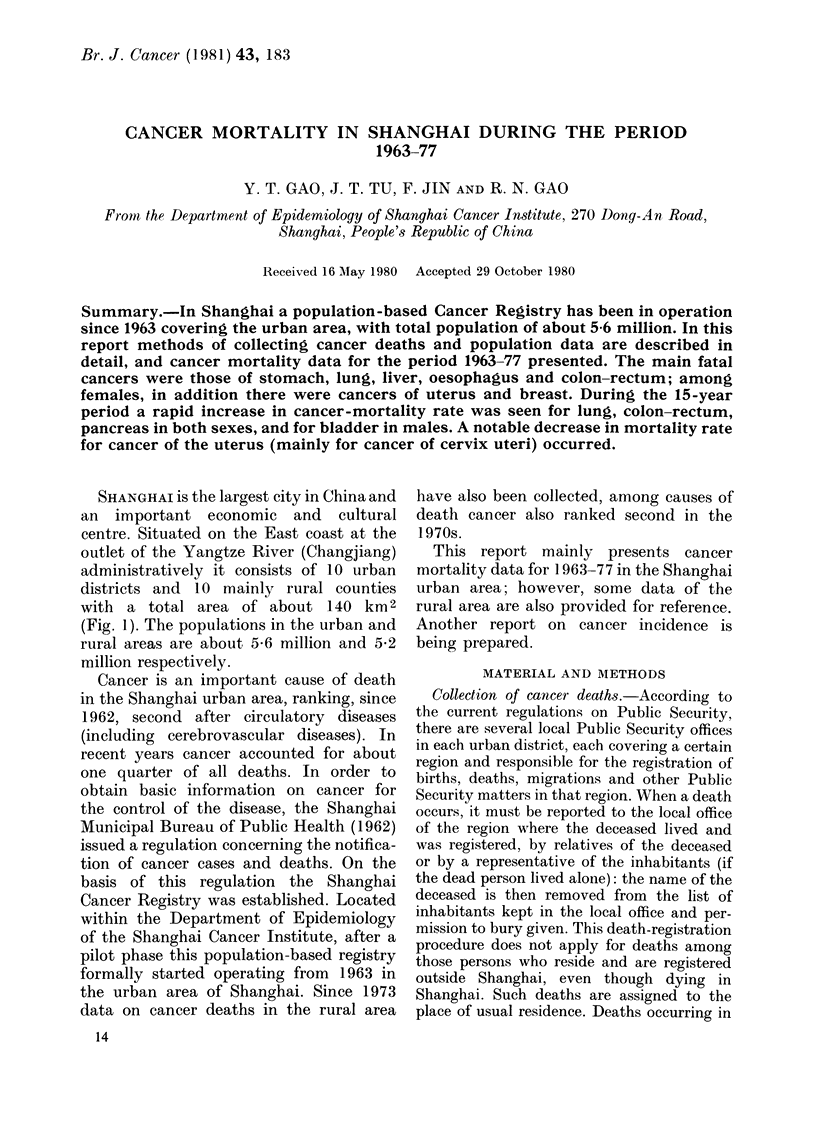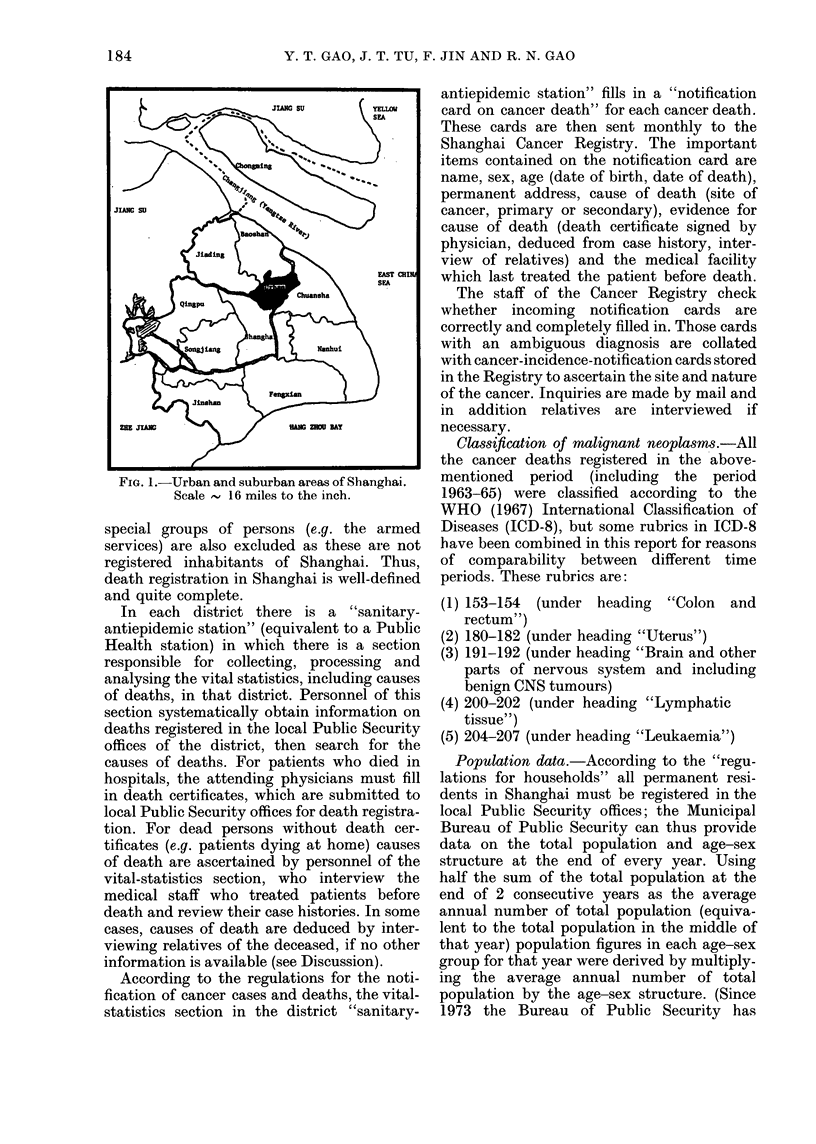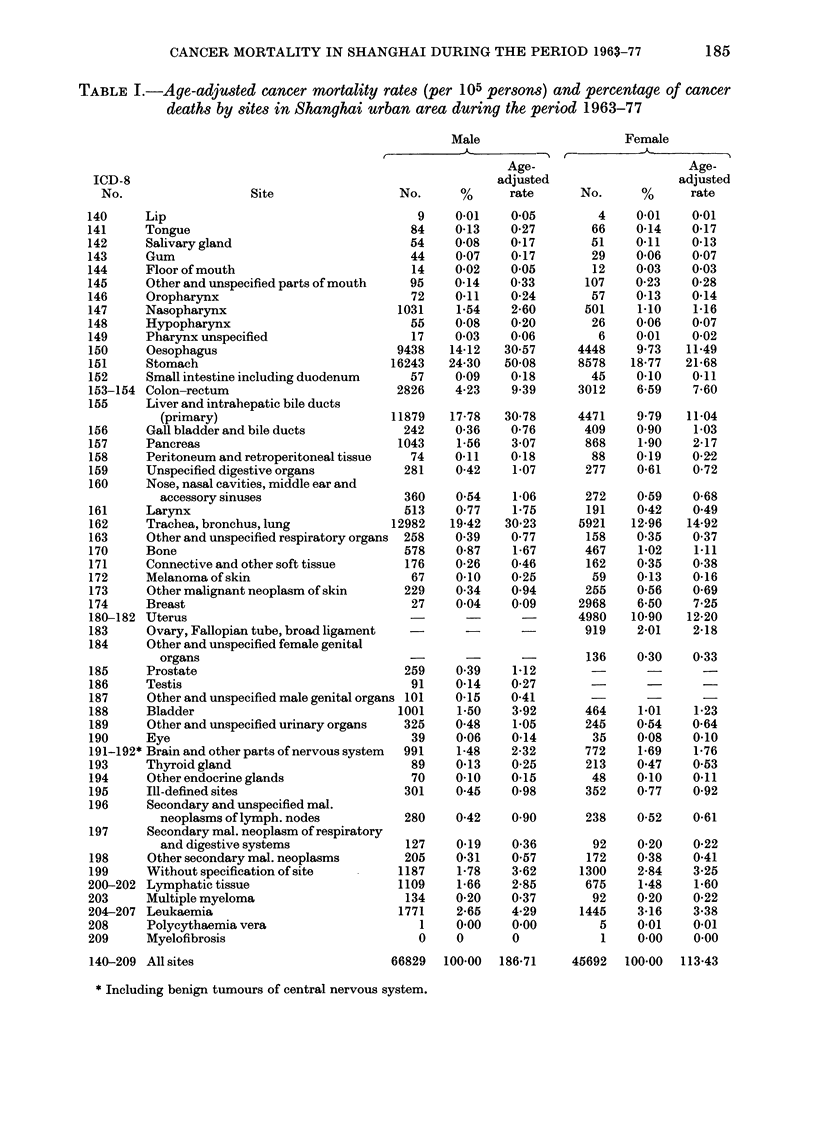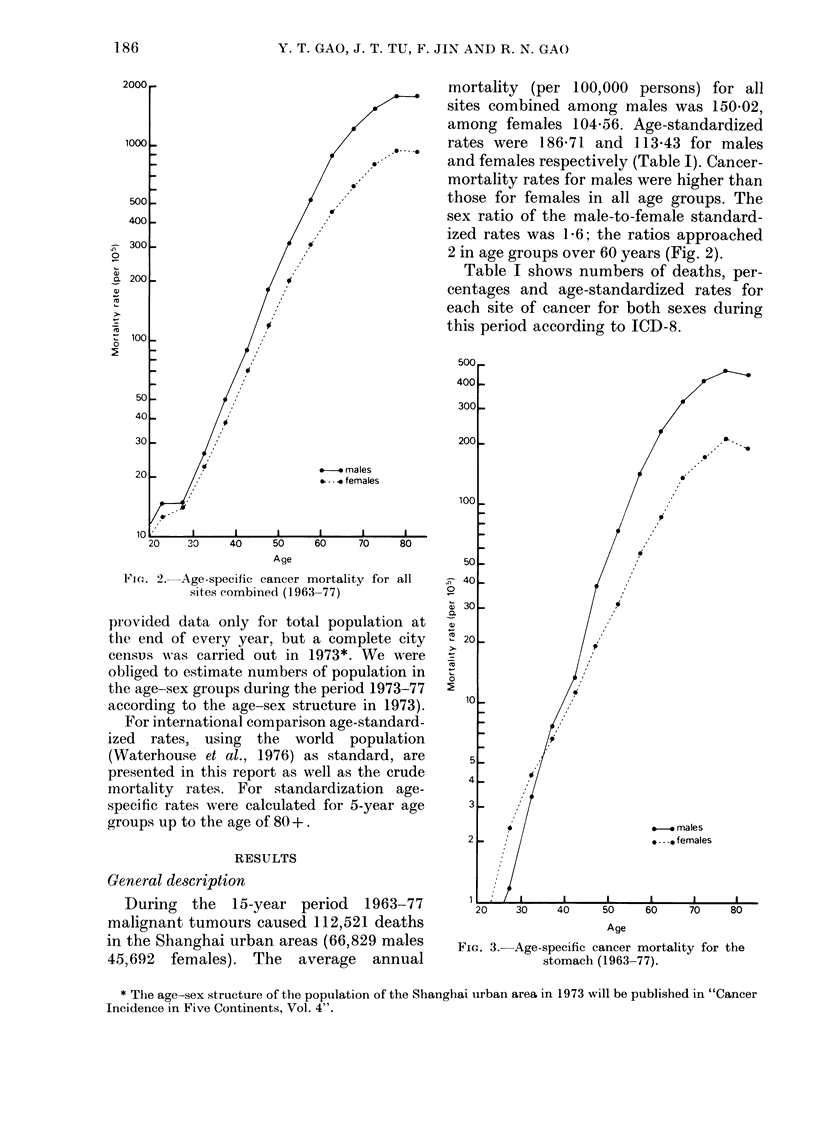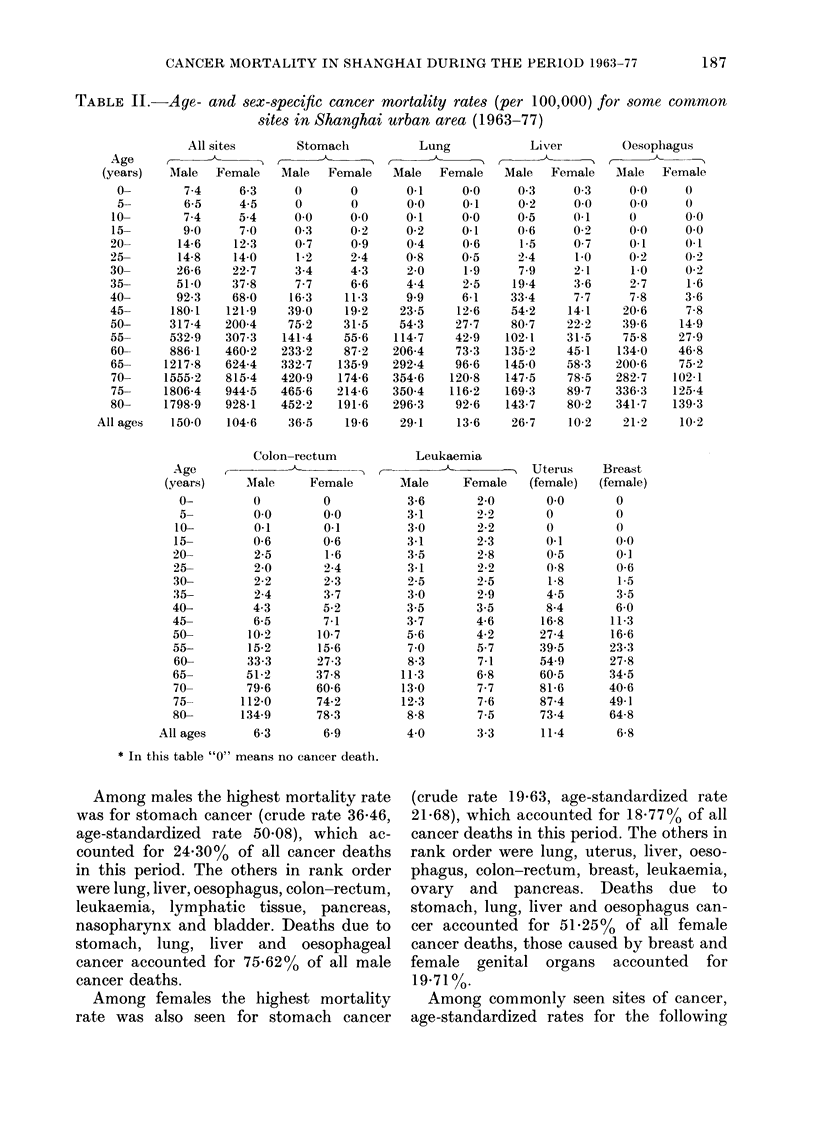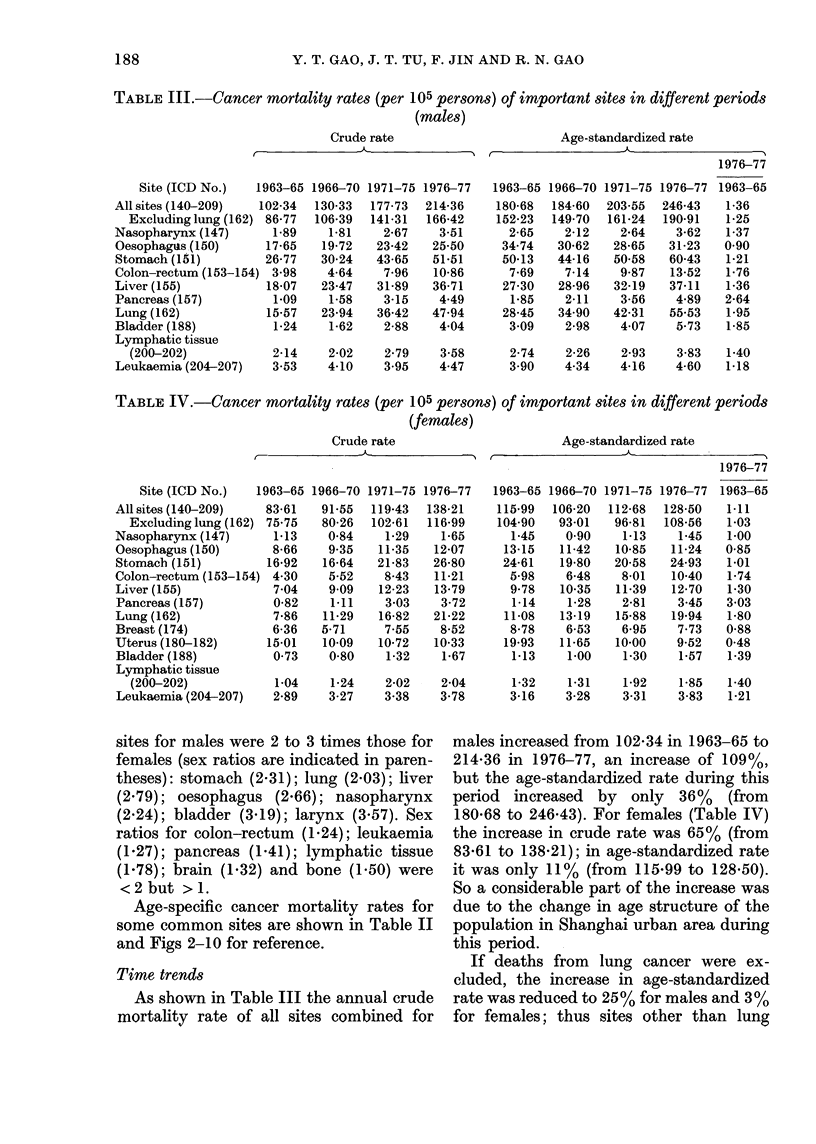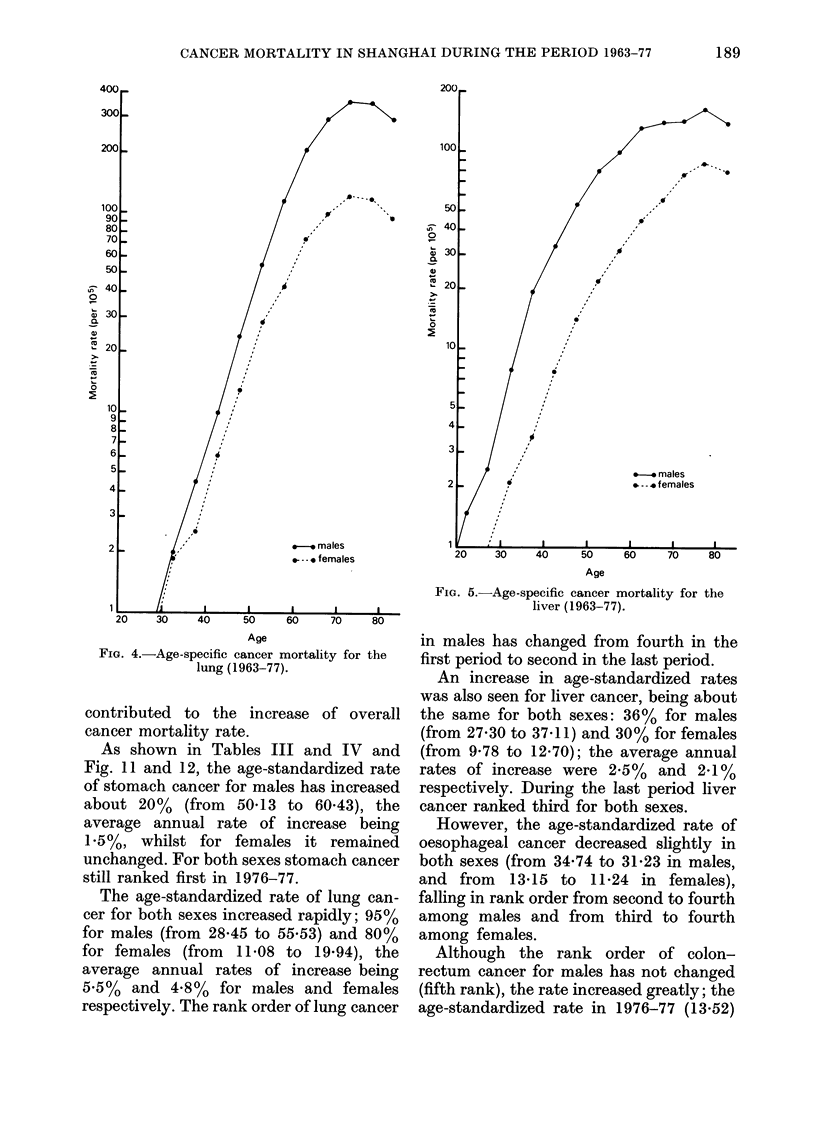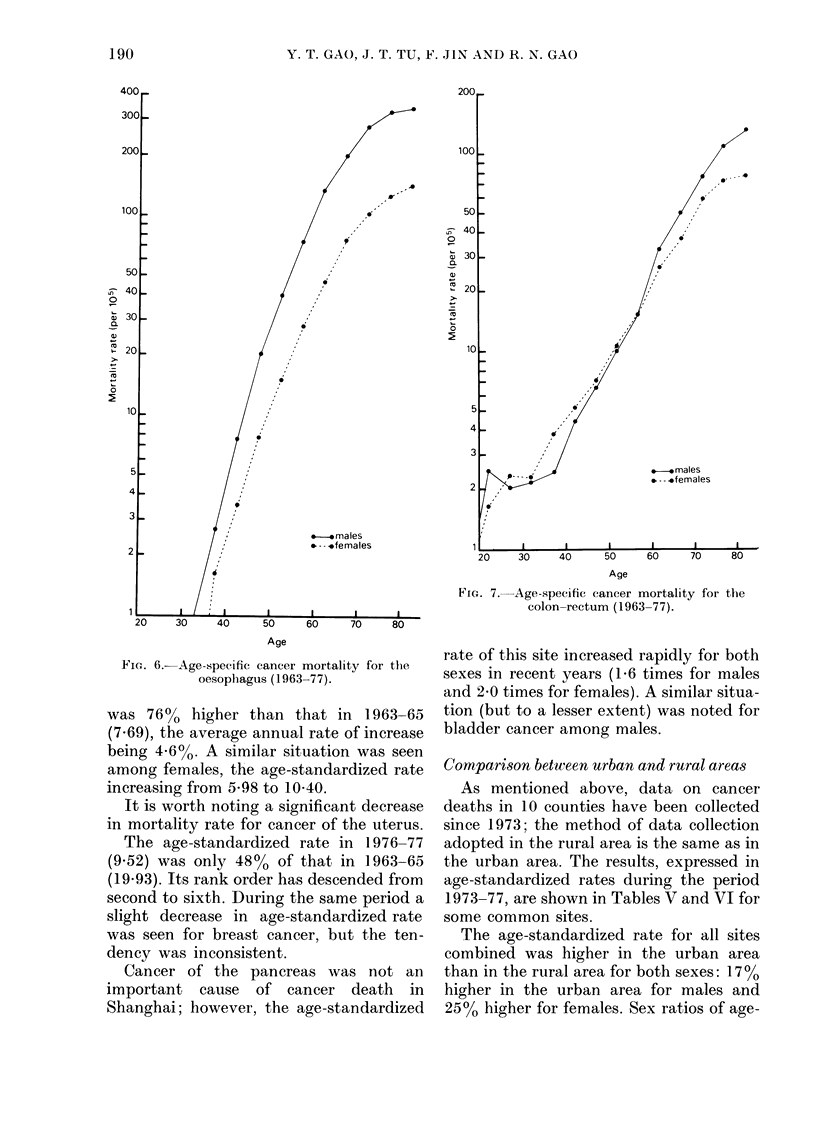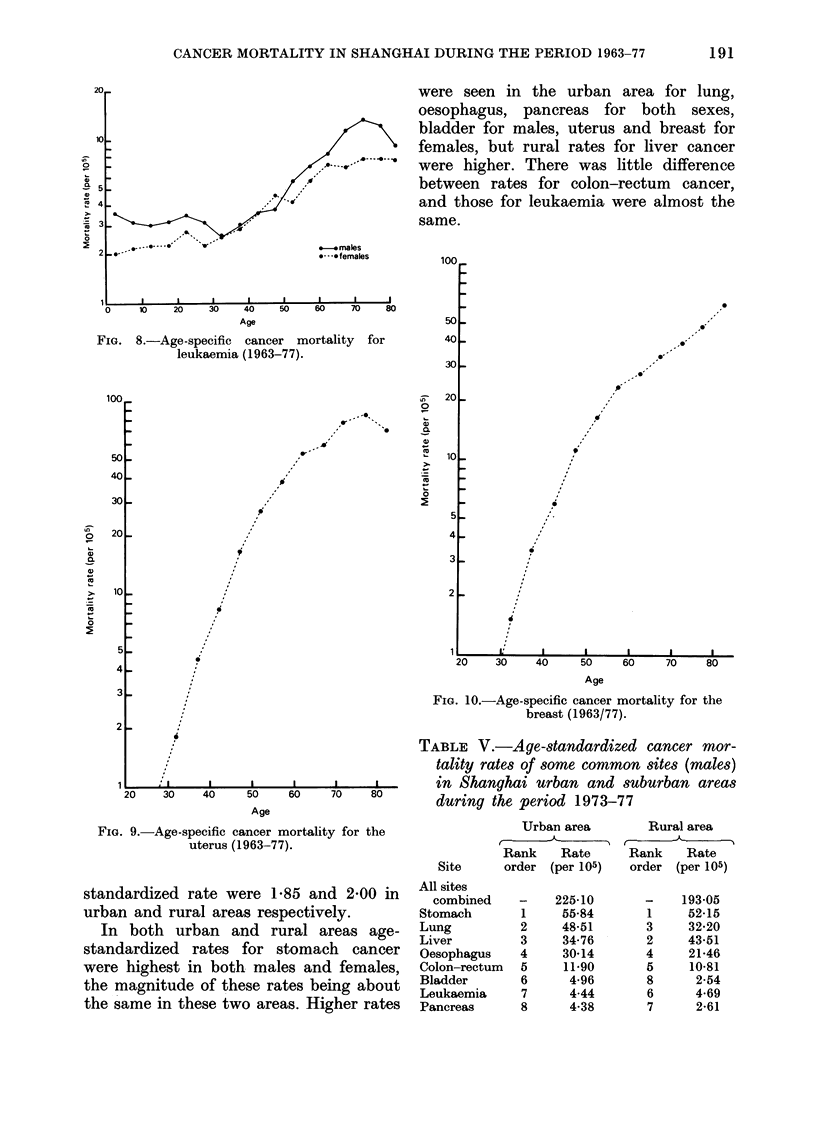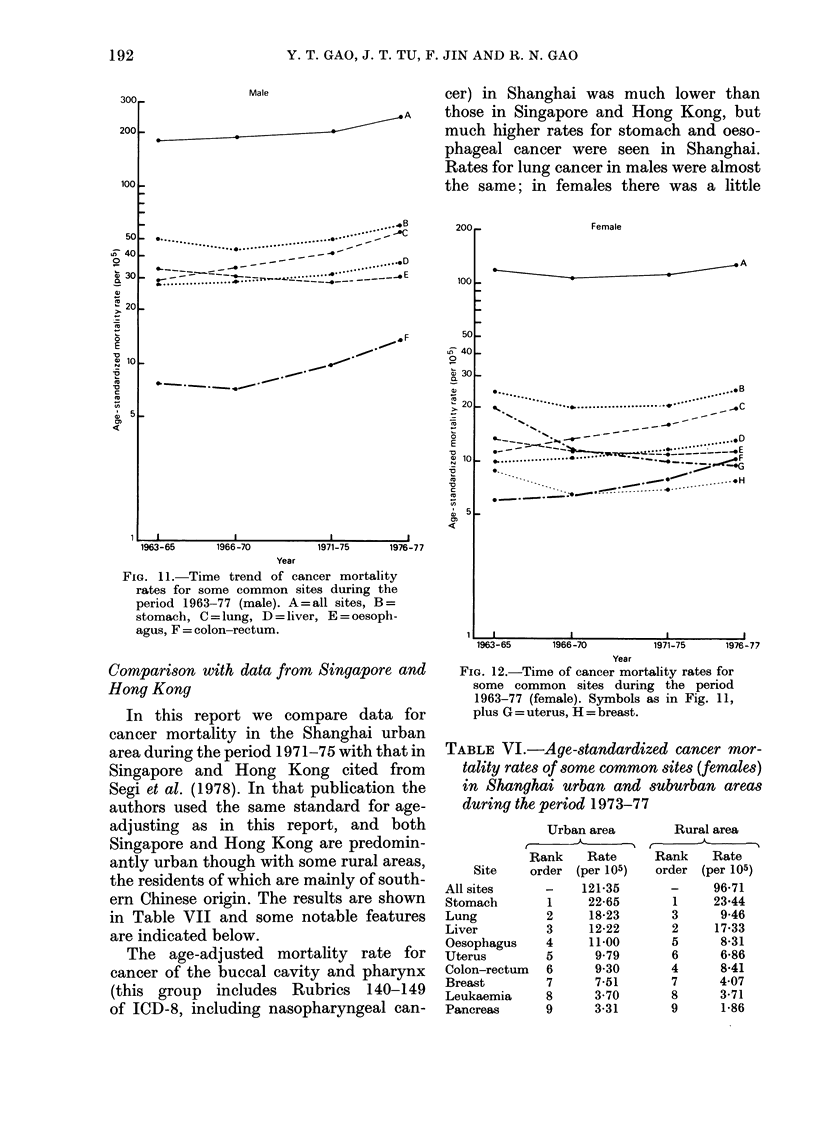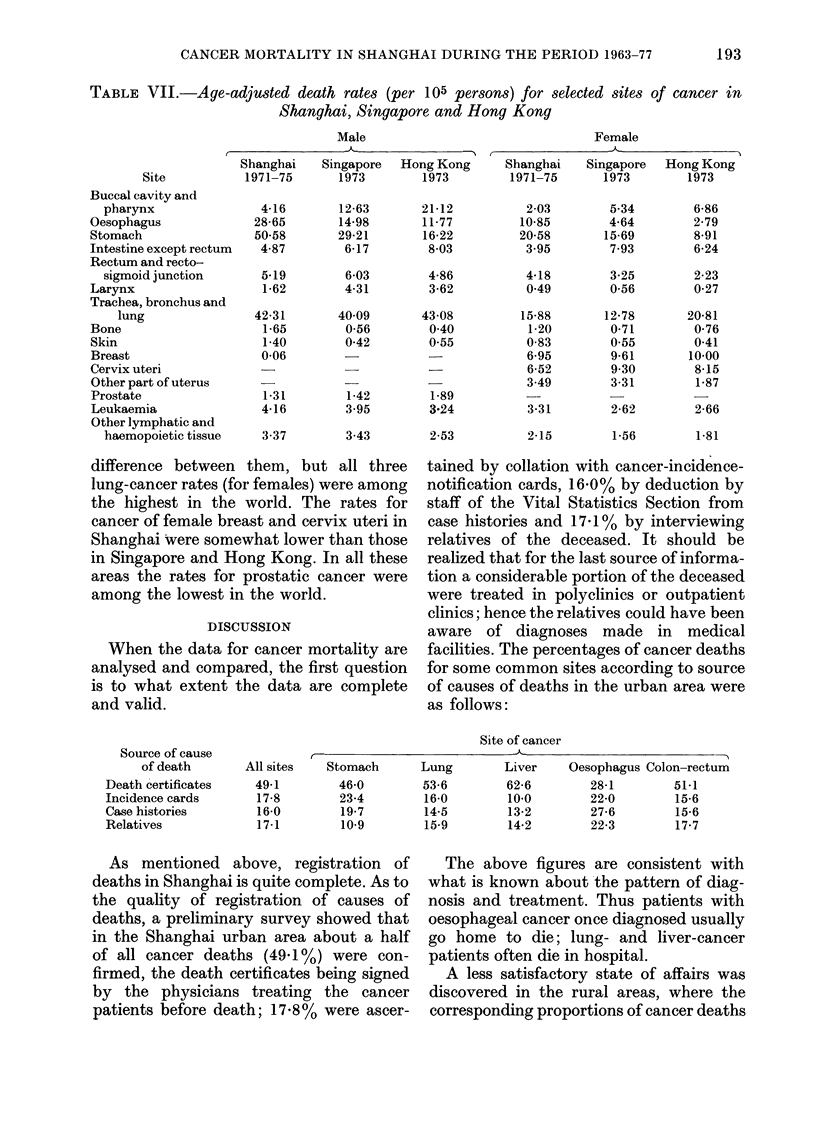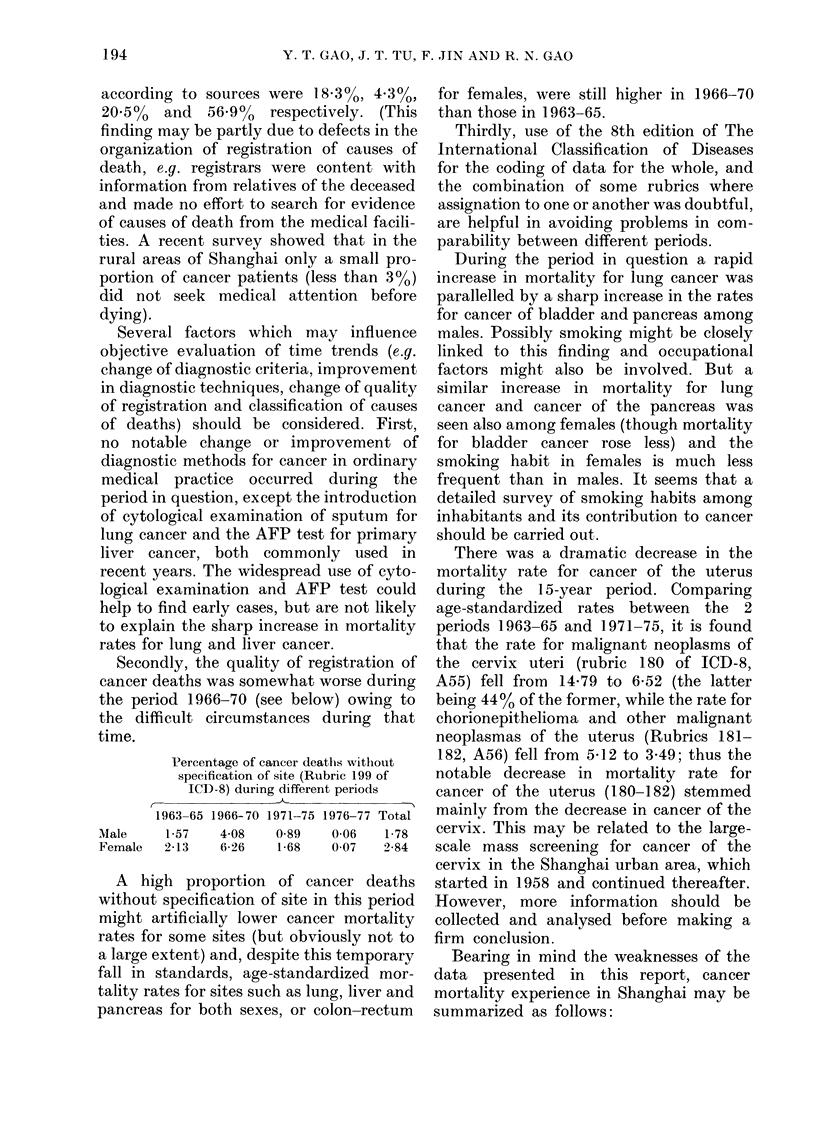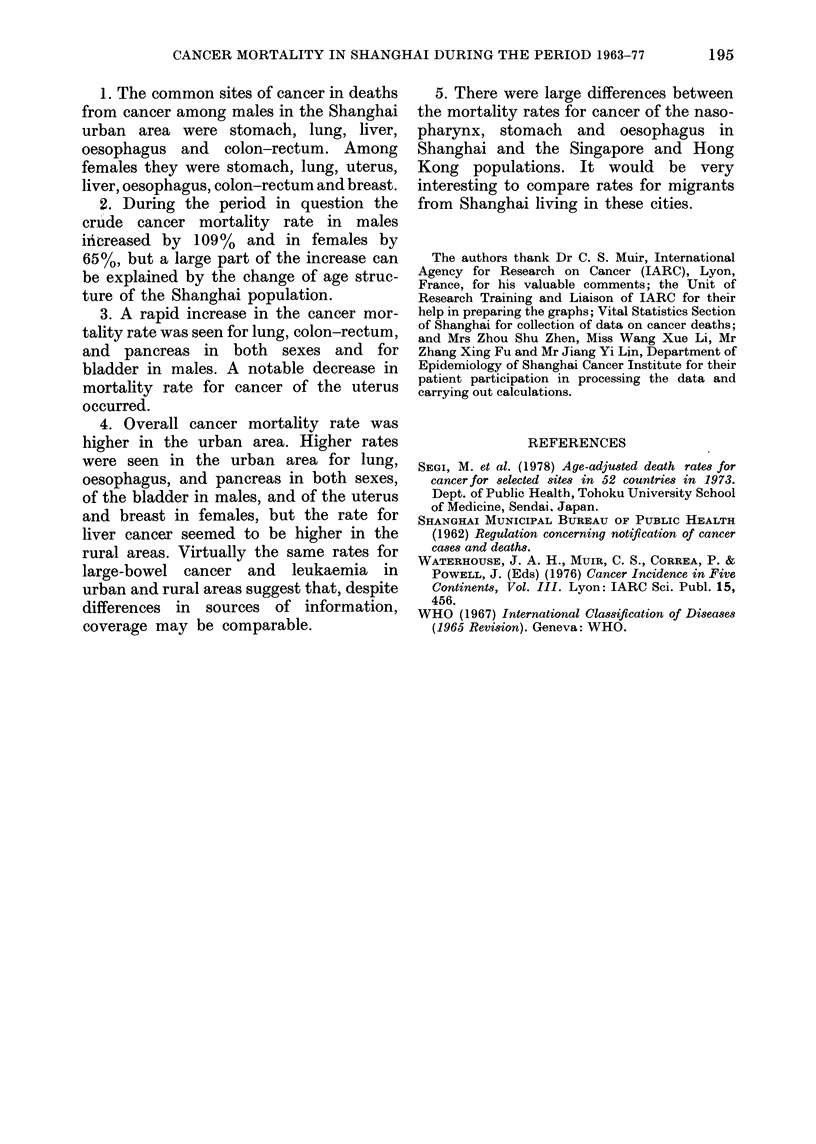# Cancer mortality in Shanghai during the period 1963-77.

**DOI:** 10.1038/bjc.1981.27

**Published:** 1981-02

**Authors:** Y. T. Gao, J. T. Tu, F. Jin, R. N. Gao

## Abstract

In Shanghai a population-based Cancer Registry has been in operation since 1963 covering the urban area, with total population of about 5.6 million. In this report methods of collecting cancer deaths and population data are described in detail, and cancer mortality data for the period 1963-77 presented. The main fatal cancers were those of stomach, lung, liver, oesophagus and colon-rectum; among females, in addition there were cancers of uterus and breast. During the 15-year period a rapid increase in cancer-mortality rate was seen for lung, colon-rectum, pancreas in both sexes, and for bladder in males. A notable decrease in mortality rate for cancer of the uterus (mainly for cancer of cervix uteri) occurred.


*Br. J. Cancer* (1981) **43**, 183

### CANCER MORTALITY IN SHANGHAI DURING THE PERIOD 1963–77

Y. T. GAO, J. T. TU, F. JIN AND R. N. GAO

*From the Department of Epidemiology of Shanghai Cancer Institute,* 270 *Dong-An Road, Shanghai, People's Republic of China*

Received 16 May 1980 Accepted 29 October 1980

**Summary.—In Shanghai a population-based Cancer Registry has been in operation since 1963 covering the urban area, with total population of about 5·6 million. In this report methods of collecting cancer deaths and population data are described in detail, and cancer mortality data for the period 1963–77 presented. The main fatal cancers were those of stomach, lung, liver, oesophagus and colon–rectum; among females, in addition there were cancers of uterus and breast. During the 15-year period a rapid increase in cancer-mortality rate was seen for lung, colon–rectum, pancreas in both sexes, and for bladder in males. A notable decrease in mortality rate for cancer of the uterus (mainly for cancer of cervix uteri) occurred.**

SHANGHAI is the largest city in China and an important economic and cultural centre. Situated on the East coast at the outlet of the Yangtze River (Changjiang) administratively it consists of 10 urban districts and 10 mainly rural counties with a total area of about 140 km$^2$ (Fig. 1). The populations in the urban and rural areas are about 5·6 million and 5·2 million respectively.

Cancer is an important cause of death in the Shanghai urban area, ranking, since 1962, second after circulatory diseases (including cerebrovascular diseases). In recent years cancer accounted for about one quarter of all deaths. In order to obtain basic information on cancer for the control of the disease, the Shanghai Municipal Bureau of Public Health (1962) issued a regulation concerning the notification of cancer cases and deaths. On the basis of this regulation the Shanghai Cancer Registry was established. Located within the Department of Epidemiology of the Shanghai Cancer Institute, after a pilot phase this population-based registry formally started operating from 1963 in the urban area of Shanghai. Since 1973 data on cancer deaths in the rural area have also been collected, among causes of death cancer also ranked second in the 1970s.

This report mainly presents cancer mortality data for 1963–77 in the Shanghai urban area; however, some data of the rural area are also provided for reference. Another report on cancer incidence is being prepared.

### MATERIAL AND METHODS

*Collection of cancer deaths.*—According to the current regulations on Public Security, there are several local Public Security offices in each urban district, each covering a certain region and responsible for the registration of births, deaths, migrations and other Public Security matters in that region. When a death occurs, it must be reported to the local office of the region where the deceased lived and was registered, by relatives of the deceased or by a representative of the inhabitants (if the dead person lived alone): the name of the deceased is then removed from the list of inhabitants kept in the local office and permission to bury given. This death-registration procedure does not apply for deaths among those persons who reside and are registered outside Shanghai, even though dying in Shanghai. Such deaths are assigned to the place of usual residence. Deaths occurring in

FIG. 1.—Urban and suburban areas of Shanghai. Scale ~ 16 miles to the inch.

special groups of persons (*e.g.* the armed services) are also excluded as these are not registered inhabitants of Shanghai. Thus, death registration in Shanghai is well-defined and quite complete.

In each district there is a "sanitary-antiepidemic station" (equivalent to a Public Health station) in which there is a section responsible for collecting, processing and analysing the vital statistics, including causes of deaths, in that district. Personnel of this section systematically obtain information on deaths registered in the local Public Security offices of the district, then search for the causes of deaths. For patients who died in hospitals, the attending physicians must fill in death certificates, which are submitted to local Public Security offices for death registration. For dead persons without death certificates (*e.g.* patients dying at home) causes of death are ascertained by personnel of the vital-statistics section, who interview the medical staff who treated patients before death and review their case histories. In some cases, causes of death are deduced by interviewing relatives of the deceased, if no other information is available (see Discussion).

According to the regulations for the notification of cancer cases and deaths, the vital-statistics section in the district "sanitary-antiepidemic station" fills in a "notification card on cancer death" for each cancer death. These cards are then sent monthly to the Shanghai Cancer Registry. The important items contained on the notification card are name, sex, age (date of birth, date of death), permanent address, cause of death (site of cancer, primary or secondary), evidence for cause of death (death certificate signed by physician, deduced from case history, interview of relatives) and the medical facility which last treated the patient before death.

The staff of the Cancer Registry check whether incoming notification cards are correctly and completely filled in. Those cards with an ambiguous diagnosis are collated with cancer-incidence-notification cards stored in the Registry to ascertain the site and nature of the cancer. Inquiries are made by mail and in addition relatives are interviewed if necessary.

*Classification of malignant neoplasms.*—All the cancer deaths registered in the above-mentioned period (including the period 1963–65) were classified according to the WHO (1967) International Classification of Diseases (ICD-8), but some rubrics in ICD-8 have been combined in this report for reasons of comparability between different time periods. These rubrics are:

(1) 153–154 (under heading "Colon and rectum")
(2) 180–182 (under heading "Uterus")
(3) 191–192 (under heading "Brain and other parts of nervous system and including benign CNS tumours)
(4) 200–202 (under heading "Lymphatic tissue")
(5) 204–207 (under heading "Leukaemia")

*Population data.*—According to the "regulations for households" all permanent residents in Shanghai must be registered in the local Public Security offices; the Municipal Bureau of Public Security can thus provide data on the total population and age–sex structure at the end of every year. Using half the sum of the total population at the end of 2 consecutive years as the average annual number of total population (equivalent to the total population in the middle of that year) population figures in each age–sex group for that year were derived by multiplying the average annual number of total population by the age–sex structure. (Since 1973 the Bureau of Public Security has

TABLE I.—*Age-adjusted cancer mortality rates (per* $10^5$ *persons) and percentage of cancer deaths by sites in Shanghai urban area during the period* 1963–77

| ICD-8 No. | Site | Male No. | Male % | Male Age-adjusted rate | Female No. | Female % | Female Age-adjusted rate |
|---|---|---|---|---|---|---|---|
| 140 | Lip | 9 | 0·01 | 0·05 | 4 | 0·01 | 0·01 |
| 141 | Tongue | 84 | 0·13 | 0·27 | 66 | 0·14 | 0·17 |
| 142 | Salivary gland | 54 | 0·08 | 0·17 | 51 | 0·11 | 0·13 |
| 143 | Gum | 44 | 0·07 | 0·17 | 29 | 0·06 | 0·07 |
| 144 | Floor of mouth | 14 | 0·02 | 0·05 | 12 | 0·03 | 0·03 |
| 145 | Other and unspecified parts of mouth | 95 | 0·14 | 0·33 | 107 | 0·23 | 0·28 |
| 146 | Oropharynx | 72 | 0·11 | 0·24 | 57 | 0·13 | 0·14 |
| 147 | Nasopharynx | 1031 | 1·54 | 2·60 | 501 | 1·10 | 1·16 |
| 148 | Hypopharynx | 55 | 0·08 | 0·20 | 26 | 0·06 | 0·07 |
| 149 | Pharynx unspecified | 17 | 0·03 | 0·06 | 6 | 0·01 | 0·02 |
| 150 | Oesophagus | 9438 | 14·12 | 30·57 | 4448 | 9·73 | 11·49 |
| 151 | Stomach | 16243 | 24·30 | 50·08 | 8578 | 18·77 | 21·68 |
| 152 | Small intestine including duodenum | 57 | 0·09 | 0·18 | 45 | 0·10 | 0·11 |
| 153–154 | Colon–rectum | 2826 | 4·23 | 9·39 | 3012 | 6·59 | 7·60 |
| 155 | Liver and intrahepatic bile ducts (primary) | 11879 | 17·78 | 30·78 | 4471 | 9·79 | 11·04 |
| 156 | Gall bladder and bile ducts | 242 | 0·36 | 0·76 | 409 | 0·90 | 1·03 |
| 157 | Pancreas | 1043 | 1·56 | 3·07 | 868 | 1·90 | 2·17 |
| 158 | Peritoneum and retroperitoneal tissue | 74 | 0·11 | 0·18 | 88 | 0·19 | 0·22 |
| 159 | Unspecified digestive organs | 281 | 0·42 | 1·07 | 277 | 0·61 | 0·72 |
| 160 | Nose, nasal cavities, middle ear and accessory sinuses | 360 | 0·54 | 1·06 | 272 | 0·59 | 0·68 |
| 161 | Larynx | 513 | 0·77 | 1·75 | 191 | 0·42 | 0·49 |
| 162 | Trachea, bronchus, lung | 12982 | 19·42 | 30·23 | 5921 | 12·96 | 14·92 |
| 163 | Other and unspecified respiratory organs | 258 | 0·39 | 0·77 | 158 | 0·35 | 0·37 |
| 170 | Bone | 578 | 0·87 | 1·67 | 467 | 1·02 | 1·11 |
| 171 | Connective and other soft tissue | 176 | 0·26 | 0·46 | 162 | 0·35 | 0·38 |
| 172 | Melanoma of skin | 67 | 0·10 | 0·25 | 59 | 0·13 | 0·16 |
| 173 | Other malignant neoplasm of skin | 229 | 0·34 | 0·94 | 255 | 0·56 | 0·69 |
| 174 | Breast | 27 | 0·04 | 0·09 | 2968 | 6·50 | 7·25 |
| 180–182 | Uterus | — | — | — | 4980 | 10·90 | 12·20 |
| 183 | Ovary, Fallopian tube, broad ligament | — | — | — | 919 | 2·01 | 2·18 |
| 184 | Other and unspecified female genital organs | — | — | — | 136 | 0·30 | 0·33 |
| 185 | Prostate | 259 | 0·39 | 1·12 | — | — | — |
| 186 | Testis | 91 | 0·14 | 0·27 | — | — | — |
| 187 | Other and unspecified male genital organs | 101 | 0·15 | 0·41 | — | — | — |
| 188 | Bladder | 1001 | 1·50 | 3·92 | 464 | 1·01 | 1·23 |
| 189 | Other and unspecified urinary organs | 325 | 0·48 | 1·05 | 245 | 0·54 | 0·64 |
| 190 | Eye | 39 | 0·06 | 0·14 | 35 | 0·08 | 0·10 |
| 191–192* | Brain and other parts of nervous system | 991 | 1·48 | 2·32 | 772 | 1·69 | 1·76 |
| 193 | Thyroid gland | 89 | 0·13 | 0·25 | 213 | 0·47 | 0·53 |
| 194 | Other endocrine glands | 70 | 0·10 | 0·15 | 48 | 0·10 | 0·11 |
| 195 | Ill-defined sites | 301 | 0·45 | 0·98 | 352 | 0·77 | 0·92 |
| 196 | Secondary and unspecified mal. neoplasms of lymph. nodes | 280 | 0·42 | 0·90 | 238 | 0·52 | 0·61 |
| 197 | Secondary mal. neoplasm of respiratory and digestive systems | 127 | 0·19 | 0·36 | 92 | 0·20 | 0·22 |
| 198 | Other secondary mal. neoplasms | 205 | 0·31 | 0·57 | 172 | 0·38 | 0·41 |
| 199 | Without specification of site | 1187 | 1·78 | 3·62 | 1300 | 2·84 | 3·25 |
| 200–202 | Lymphatic tissue | 1109 | 1·66 | 2·85 | 675 | 1·48 | 1·60 |
| 203 | Multiple myeloma | 134 | 0·20 | 0·37 | 92 | 0·20 | 0·22 |
| 204–207 | Leukaemia | 1771 | 2·65 | 4·29 | 1445 | 3·16 | 3·38 |
| 208 | Polycythaemia vera | 1 | 0·00 | 0·00 | 5 | 0·01 | 0·01 |
| 209 | Myelofibrosis | 0 | 0 | 0 | 1 | 0·00 | 0·00 |
| 140–209 | All sites | 66829 | 100·00 | 186·71 | 45692 | 100·00 | 113·43 |

* Including benign tumours of central nervous system.

FIG. 2.—Age-specific cancer mortality for all sites combined (1963–77)

provided data only for total population at the end of every year, but a complete city census was carried out in 1973*. We were obliged to estimate numbers of population in the age–sex groups during the period 1973–77 according to the age–sex structure in 1973).

For international comparison age-standardized rates, using the world population (Waterhouse *et al.*, 1976) as standard, are presented in this report as well as the crude mortality rates. For standardization age-specific rates were calculated for 5-year age groups up to the age of 80+.

### RESULTS

### *General description*

During the 15-year period 1963–77 malignant tumours caused 112,521 deaths in the Shanghai urban areas (66,829 males 45,692 females). The average annual mortality (per 100,000 persons) for all sites combined among males was 150·02, among females 104·56. Age-standardized rates were 186·71 and 113·43 for males and females respectively (Table I). Cancer-mortality rates for males were higher than those for females in all age groups. The sex ratio of the male-to-female standardized rates was 1·6; the ratios approached 2 in age groups over 60 years (Fig. 2).

Table I shows numbers of deaths, percentages and age-standardized rates for each site of cancer for both sexes during this period according to ICD-8.

FIG. 3.—Age-specific cancer mortality for the stomach (1963–77).

* The age–sex structure of the population of the Shanghai urban area in 1973 will be published in "Cancer Incidence in Five Continents, Vol. 4".

TABLE II.—*Age- and sex-specific cancer mortality rates (per* 100,000) *for some common sites in Shanghai urban area* (1963–77)

| Age (years) | All sites | | Stomach | | Lung | | Liver | | Oesophagus | |
|---|---|---|---|---|---|---|---|---|---|---|
| | Male | Female | Male | Female | Male | Female | Male | Female | Male | Female |
| 0– | 7·4 | 6·3 | 0 | 0 | 0·1 | 0·0 | 0·3 | 0·3 | 0·0 | 0 |
| 5– | 6·5 | 4·5 | 0 | 0 | 0·0 | 0·1 | 0·2 | 0·0 | 0·0 | 0 |
| 10– | 7·4 | 5·4 | 0·0 | 0·0 | 0·1 | 0·0 | 0·5 | 0·1 | 0 | 0·0 |
| 15– | 9·0 | 7·0 | 0·3 | 0·2 | 0·2 | 0·1 | 0·6 | 0·2 | 0·0 | 0·0 |
| 20– | 14·6 | 12·3 | 0·7 | 0·9 | 0·4 | 0·6 | 1·5 | 0·7 | 0·1 | 0·1 |
| 25– | 14·8 | 14·0 | 1·2 | 2·4 | 0·8 | 0·5 | 2·4 | 1·0 | 0·2 | 0·2 |
| 30– | 26·6 | 22·7 | 3·4 | 4·3 | 2·0 | 1·9 | 7·9 | 2·1 | 1·0 | 0·2 |
| 35– | 51·0 | 37·8 | 7·7 | 6·6 | 4·4 | 2·5 | 19·4 | 3·6 | 2·7 | 1·6 |
| 40– | 92·3 | 68·0 | 16·3 | 11·3 | 9·9 | 6·1 | 33·4 | 7·7 | 7·8 | 3·6 |
| 45– | 180·1 | 121·9 | 39·0 | 19·2 | 23·5 | 12·6 | 54·2 | 14·1 | 20·6 | 7·8 |
| 50– | 317·4 | 200·4 | 75·2 | 31·5 | 54·3 | 27·7 | 80·7 | 22·2 | 39·6 | 14·9 |
| 55– | 532·9 | 307·3 | 141·4 | 55·6 | 114·7 | 42·9 | 102·1 | 31·5 | 75·8 | 27·9 |
| 60– | 886·1 | 460·2 | 233·2 | 87·2 | 206·4 | 73·3 | 135·2 | 45·1 | 134·0 | 46·8 |
| 65– | 1217·8 | 624·4 | 332·7 | 135·9 | 292·4 | 96·6 | 145·0 | 58·3 | 200·6 | 75·2 |
| 70– | 1555·2 | 815·4 | 420·9 | 174·6 | 354·6 | 120·8 | 147·5 | 78·5 | 282·7 | 102·1 |
| 75– | 1806·4 | 944·5 | 465·6 | 214·6 | 350·4 | 116·2 | 169·3 | 89·7 | 336·3 | 125·4 |
| 80– | 1798·9 | 928·1 | 452·2 | 191·6 | 296·3 | 92·6 | 143·7 | 80·2 | 341·7 | 139·3 |
| All ages | 150·0 | 104·6 | 36·5 | 19·6 | 29·1 | 13·6 | 26·7 | 10·2 | 21·2 | 10·2 |

| Age (years) | Colon–rectum | | Leukaemia | | Uterus (female) | Breast (female) |
|---|---|---|---|---|---|---|
| | Male | Female | Male | Female | | |
| 0– | 0 | 0 | 3·6 | 2·0 | 0·0 | 0 |
| 5– | 0·0 | 0·0 | 3·1 | 2·2 | 0 | 0 |
| 10– | 0·1 | 0·1 | 3·0 | 2·2 | 0 | 0 |
| 15– | 0·6 | 0·6 | 3·1 | 2·3 | 0·1 | 0·0 |
| 20– | 2·5 | 1·6 | 3·5 | 2·8 | 0·5 | 0·1 |
| 25– | 2·0 | 2·4 | 3·1 | 2·2 | 0·8 | 0·6 |
| 30– | 2·2 | 2·3 | 2·5 | 2·5 | 1·8 | 1·5 |
| 35– | 2·4 | 3·7 | 3·0 | 2·9 | 4·5 | 3·5 |
| 40– | 4·3 | 5·2 | 3·5 | 3·5 | 8·4 | 6·0 |
| 45– | 6·5 | 7·1 | 3·7 | 4·6 | 16·8 | 11·3 |
| 50– | 10·2 | 10·7 | 5·6 | 4·2 | 27·4 | 16·6 |
| 55– | 15·2 | 15·6 | 7·0 | 5·7 | 39·5 | 23·3 |
| 60– | 33·3 | 27·3 | 8·3 | 7·1 | 54·9 | 27·8 |
| 65– | 51·2 | 37·8 | 11·3 | 6·8 | 60·5 | 34·5 |
| 70– | 79·6 | 60·6 | 13·0 | 7·7 | 81·6 | 40·6 |
| 75– | 112·0 | 74·2 | 12·3 | 7·6 | 87·4 | 49·1 |
| 80– | 134·9 | 78·3 | 8·8 | 7·5 | 73·4 | 64·8 |
| All ages | 6·3 | 6·9 | 4·0 | 3·3 | 11·4 | 6·8 |

* In this table "0" means no cancer death.

Among males the highest mortality rate was for stomach cancer (crude rate 36·46, age-standardized rate 50·08), which accounted for 24·30% of all cancer deaths in this period. The others in rank order were lung, liver, oesophagus, colon–rectum, leukaemia, lymphatic tissue, pancreas, nasopharynx and bladder. Deaths due to stomach, lung, liver and oesophageal cancer accounted for 75·62% of all male cancer deaths.

Among females the highest mortality rate was also seen for stomach cancer (crude rate 19·63, age-standardized rate 21·68), which accounted for 18·77% of all cancer deaths in this period. The others in rank order were lung, uterus, liver, oesophagus, colon–rectum, breast, leukaemia, ovary and pancreas. Deaths due to stomach, lung, liver and oesophagus cancer accounted for 51·25% of all female cancer deaths, those caused by breast and female genital organs accounted for 19·71%.

Among commonly seen sites of cancer, age-standardized rates for the following

TABLE III.—*Cancer mortality rates (per* $10^5$ *persons) of important sites in different periods (males)*

| Site (ICD No.) | Crude rate 1963–65 | 1966–70 | 1971–75 | 1976–77 | Age-standardized rate 1963–65 | 1966–70 | 1971–75 | 1976–77 | 1976–77 / 1963–65 |
|---|---|---|---|---|---|---|---|---|---|
| All sites (140–209) | 102·34 | 130·33 | 177·73 | 214·36 | 180·68 | 184·60 | 203·55 | 246·43 | 1·36 |
| Excluding lung (162) | 86·77 | 106·39 | 141·31 | 166·42 | 152·23 | 149·70 | 161·24 | 190·91 | 1·25 |
| Nasopharynx (147) | 1·89 | 1·81 | 2·67 | 3·51 | 2·65 | 2·12 | 2·64 | 3·62 | 1·37 |
| Oesophagus (150) | 17·65 | 19·72 | 23·42 | 25·50 | 34·74 | 30·62 | 28·65 | 31·23 | 0·90 |
| Stomach (151) | 26·77 | 30·24 | 43·65 | 51·51 | 50·13 | 44·16 | 50·58 | 60·43 | 1·21 |
| Colon–rectum (153–154) | 3·98 | 4·64 | 7·96 | 10·86 | 7·69 | 7·14 | 9·87 | 13·52 | 1·76 |
| Liver (155) | 18·07 | 23·47 | 31·89 | 36·71 | 27·30 | 28·96 | 32·19 | 37·11 | 1·36 |
| Pancreas (157) | 1·09 | 1·58 | 3·15 | 4·49 | 1·85 | 2·11 | 3·56 | 4·89 | 2·64 |
| Lung (162) | 15·57 | 23·94 | 36·42 | 47·94 | 28·45 | 34·90 | 42·31 | 55·53 | 1·95 |
| Bladder (188) | 1·24 | 1·62 | 2·88 | 4·04 | 3·09 | 2·98 | 4·07 | 5·73 | 1·85 |
| Lymphatic tissue (200–202) | 2·14 | 2·02 | 2·79 | 3·58 | 2·74 | 2·26 | 2·93 | 3·83 | 1·40 |
| Leukaemia (204–207) | 3·53 | 4·10 | 3·95 | 4·47 | 3·90 | 4·34 | 4·16 | 4·60 | 1·18 |

TABLE IV.—*Cancer mortality rates (per* $10^5$ *persons) of important sites in different periods (females)*

| Site (ICD No.) | Crude rate 1963–65 | 1966–70 | 1971–75 | 1976–77 | Age-standardized rate 1963–65 | 1966–70 | 1971–75 | 1976–77 | 1976–77 / 1963–65 |
|---|---|---|---|---|---|---|---|---|---|
| All sites (140–209) | 83·61 | 91·55 | 119·43 | 138·21 | 115·99 | 106·20 | 112·68 | 128·50 | 1·11 |
| Excluding lung (162) | 75·75 | 80·26 | 102·61 | 116·99 | 104·90 | 93·01 | 96·81 | 108·56 | 1·03 |
| Nasopharynx (147) | 1·13 | 0·84 | 1·29 | 1·65 | 1·45 | 0·90 | 1·13 | 1·45 | 1·00 |
| Oesophagus (150) | 8·66 | 9·35 | 11·35 | 12·07 | 13·15 | 11·42 | 10·85 | 11·24 | 0·85 |
| Stomach (151) | 16·92 | 16·64 | 21·83 | 26·80 | 24·61 | 19·80 | 20·58 | 24·93 | 1·01 |
| Colon–rectum (153–154) | 4·30 | 5·52 | 8·43 | 11·21 | 5·98 | 6·48 | 8·01 | 10·40 | 1·74 |
| Liver (155) | 7·04 | 9·09 | 12·23 | 13·79 | 9·78 | 10·35 | 11·39 | 12·70 | 1·30 |
| Pancreas (157) | 0·82 | 1·11 | 3·03 | 3·72 | 1·14 | 1·28 | 2·81 | 3·45 | 3·03 |
| Lung (162) | 7·86 | 11·29 | 16·82 | 21·22 | 11·08 | 13·19 | 15·88 | 19·94 | 1·80 |
| Breast (174) | 6·36 | 5·71 | 7·55 | 8·52 | 8·78 | 6·53 | 6·95 | 7·73 | 0·88 |
| Uterus (180–182) | 15·01 | 10·09 | 10·72 | 10·33 | 19·93 | 11·65 | 10·00 | 9·52 | 0·48 |
| Bladder (188) | 0·73 | 0·80 | 1·32 | 1·67 | 1·13 | 1·00 | 1·30 | 1·57 | 1·39 |
| Lymphatic tissue (200–202) | 1·04 | 1·24 | 2·02 | 2·04 | 1·32 | 1·31 | 1·92 | 1·85 | 1·40 |
| Leukaemia (204–207) | 2·89 | 3·27 | 3·38 | 3·78 | 3·16 | 3·28 | 3·31 | 3·83 | 1·21 |

sites for males were 2 to 3 times those for females (sex ratios are indicated in parentheses): stomach (2·31); lung (2·03); liver (2·79); oesophagus (2·66); nasopharynx (2·24); bladder (3·19); larynx (3·57). Sex ratios for colon–rectum (1·24); leukaemia (1·27); pancreas (1·41); lymphatic tissue (1·78); brain (1·32) and bone (1·50) were $< 2$ but $> 1$.

Age-specific cancer mortality rates for some common sites are shown in Table II and Figs 2–10 for reference.

*Time trends*

As shown in Table III the annual crude mortality rate of all sites combined for males increased from 102·34 in 1963–65 to 214·36 in 1976–77, an increase of 109%, but the age-standardized rate during this period increased by only 36% (from 180·68 to 246·43). For females (Table IV) the increase in crude rate was 65% (from 83·61 to 138·21); in age-standardized rate it was only 11% (from 115·99 to 128·50). So a considerable part of the increase was due to the change in age structure of the population in Shanghai urban area during this period.

If deaths from lung cancer were excluded, the increase in age-standardized rate was reduced to 25% for males and 3% for females; thus sites other than lung

Fig. 4.—Age-specific cancer mortality for the lung (1963–77).

Fig. 5.—Age-specific cancer mortality for the liver (1963–77).

contributed to the increase of overall cancer mortality rate.

As shown in Tables III and IV and Fig. 11 and 12, the age-standardized rate of stomach cancer for males has increased about 20% (from 50·13 to 60·43), the average annual rate of increase being 1·5%, whilst for females it remained unchanged. For both sexes stomach cancer still ranked first in 1976–77.

The age-standardized rate of lung cancer for both sexes increased rapidly; 95% for males (from 28·45 to 55·53) and 80% for females (from 11·08 to 19·94), the average annual rates of increase being 5·5% and 4·8% for males and females respectively. The rank order of lung cancer in males has changed from fourth in the first period to second in the last period.

An increase in age-standardized rates was also seen for liver cancer, being about the same for both sexes: 36% for males (from 27·30 to 37·11) and 30% for females (from 9·78 to 12·70); the average annual rates of increase were 2·5% and 2·1% respectively. During the last period liver cancer ranked third for both sexes.

However, the age-standardized rate of oesophageal cancer decreased slightly in both sexes (from 34·74 to 31·23 in males, and from 13·15 to 11·24 in females), falling in rank order from second to fourth among males and from third to fourth among females.

Although the rank order of colon–rectum cancer for males has not changed (fifth rank), the rate increased greatly; the age-standardized rate in 1976–77 (13·52)

FIG. 6.—Age-specific cancer mortality for the oesophagus (1963–77).

FIG. 7.—Age-specific cancer mortality for the colon–rectum (1963–77).

was 76% higher than that in 1963–65 (7·69), the average annual rate of increase being 4·6%. A similar situation was seen among females, the age-standardized rate increasing from 5·98 to 10·40.

It is worth noting a significant decrease in mortality rate for cancer of the uterus. The age-standardized rate in 1976–77 (9·52) was only 48% of that in 1963–65 (19·93). Its rank order has descended from second to sixth. During the same period a slight decrease in age-standardized rate was seen for breast cancer, but the tendency was inconsistent.

Cancer of the pancreas was not an important cause of cancer death in Shanghai; however, the age-standardized rate of this site increased rapidly for both sexes in recent years (1·6 times for males and 2·0 times for females). A similar situation (but to a lesser extent) was noted for bladder cancer among males.

*Comparison between urban and rural areas*

As mentioned above, data on cancer deaths in 10 counties have been collected since 1973; the method of data collection adopted in the rural area is the same as in the urban area. The results, expressed in age-standardized rates during the period 1973–77, are shown in Tables V and VI for some common sites.

The age-standardized rate for all sites combined was higher in the urban area than in the rural area for both sexes: 17% higher in the urban area for males and 25% higher for females. Sex ratios of age-

FIG. 8.—Age-specific cancer mortality for leukaemia (1963–77).

FIG. 9.—Age-specific cancer mortality for the uterus (1963–77).

standardized rate were 1·85 and 2·00 in urban and rural areas respectively.

In both urban and rural areas age-standardized rates for stomach cancer were highest in both males and females, the magnitude of these rates being about the same in these two areas. Higher rates were seen in the urban area for lung, oesophagus, pancreas for both sexes, bladder for males, uterus and breast for females, but rural rates for liver cancer were higher. There was little difference between rates for colon–rectum cancer, and those for leukaemia were almost the same.

FIG. 10.—Age-specific cancer mortality for the breast (1963/77).

TABLE V.—*Age-standardized cancer mortality rates of some common sites (males) in Shanghai urban and suburban areas during the period* 1973–77

| | Urban area | | Rural area | |
|---|---|---|---|---|
| Site | Rank order | Rate (per $10^5$) | Rank order | Rate (per $10^5$) |
| All sites combined | – | 225·10 | – | 193·05 |
| Stomach | 1 | 55·84 | 1 | 52·15 |
| Lung | 2 | 48·51 | 3 | 32·20 |
| Liver | 3 | 34·76 | 2 | 43·51 |
| Oesophagus | 4 | 30·14 | 4 | 21·46 |
| Colon–rectum | 5 | 11·90 | 5 | 10·81 |
| Bladder | 6 | 4·96 | 8 | 2·54 |
| Leukaemia | 7 | 4·44 | 6 | 4·69 |
| Pancreas | 8 | 4·38 | 7 | 2·61 |

FIG. 11.—Time trend of cancer mortality rates for some common sites during the period 1963–77 (male). A = all sites, B = stomach, C = lung, D = liver, E = oesophagus, F = colon–rectum.

### *Comparison with data from Singapore and Hong Kong*

In this report we compare data for cancer mortality in the Shanghai urban area during the period 1971–75 with that in Singapore and Hong Kong cited from Segi *et al.* (1978). In that publication the authors used the same standard for age-adjusting as in this report, and both Singapore and Hong Kong are predominantly urban though with some rural areas, the residents of which are mainly of southern Chinese origin. The results are shown in Table VII and some notable features are indicated below.

The age-adjusted mortality rate for cancer of the buccal cavity and pharynx (this group includes Rubrics 140–149 of ICD-8, including nasopharyngeal cancer) in Shanghai was much lower than those in Singapore and Hong Kong, but much higher rates for stomach and oesophageal cancer were seen in Shanghai. Rates for lung cancer in males were almost the same; in females there was a little

FIG. 12.—Time of cancer mortality rates for some common sites during the period 1963–77 (female). Symbols as in Fig. 11, plus G = uterus, H = breast.

TABLE VI.—*Age-standardized cancer mortality rates of some common sites (females) in Shanghai urban and suburban areas during the period* 1973–77

| Site | Urban area Rank order | Urban area Rate (per $10^5$) | Rural area Rank order | Rural area Rate (per $10^5$) |
|---|---|---|---|---|
| All sites | – | 121·35 | – | 96·71 |
| Stomach | 1 | 22·65 | 1 | 23·44 |
| Lung | 2 | 18·23 | 3 | 9·46 |
| Liver | 3 | 12·22 | 2 | 17·33 |
| Oesophagus | 4 | 11·00 | 5 | 8·31 |
| Uterus | 5 | 9·79 | 6 | 6·86 |
| Colon–rectum | 6 | 9·30 | 4 | 8·41 |
| Breast | 7 | 7·51 | 7 | 4·07 |
| Leukaemia | 8 | 3·70 | 8 | 3·71 |
| Pancreas | 9 | 3·31 | 9 | 1·86 |

TABLE VII.—*Age-adjusted death rates (per* $10^5$ *persons) for selected sites of cancer in Shanghai, Singapore and Hong Kong*

| Site | Male: Shanghai 1971–75 | Male: Singapore 1973 | Male: Hong Kong 1973 | Female: Shanghai 1971–75 | Female: Singapore 1973 | Female: Hong Kong 1973 |
|---|---|---|---|---|---|---|
| Buccal cavity and pharynx | 4·16 | 12·63 | 21·12 | 2·03 | 5·34 | 6·86 |
| Oesophagus | 28·65 | 14·98 | 11·77 | 10·85 | 4·64 | 2·79 |
| Stomach | 50·58 | 29·21 | 16·22 | 20·58 | 15·69 | 8·91 |
| Intestine except rectum | 4·87 | 6·17 | 8·03 | 3·95 | 7·93 | 6·24 |
| Rectum and recto–sigmoid junction | 5·19 | 6·03 | 4·86 | 4·18 | 3·25 | 2·23 |
| Larynx | 1·62 | 4·31 | 3·62 | 0·49 | 0·56 | 0·27 |
| Trachea, bronchus and lung | 42·31 | 40·09 | 43·08 | 15·88 | 12·78 | 20·81 |
| Bone | 1·65 | 0·56 | 0·40 | 1·20 | 0·71 | 0·76 |
| Skin | 1·40 | 0·42 | 0·55 | 0·83 | 0·55 | 0·41 |
| Breast | 0·06 | — | — | 6·95 | 9·61 | 10·00 |
| Cervix uteri | — | — | — | 6·52 | 9·30 | 8·15 |
| Other part of uterus | — | — | — | 3·49 | 3·31 | 1·87 |
| Prostate | 1·31 | 1·42 | 1·89 | — | — | — |
| Leukaemia | 4·16 | 3·95 | 3·24 | 3·31 | 2·62 | 2·66 |
| Other lymphatic and haemopoietic tissue | 3·37 | 3·43 | 2·53 | 2·15 | 1·56 | 1·81 |

difference between them, but all three lung-cancer rates (for females) were among the highest in the world. The rates for cancer of female breast and cervix uteri in Shanghai were somewhat lower than those in Singapore and Hong Kong. In all these areas the rates for prostatic cancer were among the lowest in the world.

### DISCUSSION

When the data for cancer mortality are analysed and compared, the first question is to what extent the data are complete and valid.

As mentioned above, registration of deaths in Shanghai is quite complete. As to the quality of registration of causes of deaths, a preliminary survey showed that in the Shanghai urban area about a half of all cancer deaths (49·1%) were confirmed, the death certificates being signed by the physicians treating the cancer patients before death; 17·8% were ascertained by collation with cancer-incidence-notification cards, 16·0% by deduction by staff of the Vital Statistics Section from case histories and 17·1% by interviewing relatives of the deceased. It should be realized that for the last source of information a considerable portion of the deceased were treated in polyclinics or outpatient clinics; hence the relatives could have been aware of diagnoses made in medical facilities. The percentages of cancer deaths for some common sites according to source of causes of deaths in the urban area were as follows:

| Source of cause of death | All sites | Site of cancer: Stomach | Lung | Liver | Oesophagus | Colon–rectum |
|---|---|---|---|---|---|---|
| Death certificates | 49·1 | 46·0 | 53·6 | 62·6 | 28·1 | 51·1 |
| Incidence cards | 17·8 | 23·4 | 16·0 | 10·0 | 22·0 | 15·6 |
| Case histories | 16·0 | 19·7 | 14·5 | 13·2 | 27·6 | 15·6 |
| Relatives | 17·1 | 10·9 | 15·9 | 14·2 | 22·3 | 17·7 |

The above figures are consistent with what is known about the pattern of diagnosis and treatment. Thus patients with oesophageal cancer once diagnosed usually go home to die; lung- and liver-cancer patients often die in hospital.

A less satisfactory state of affairs was discovered in the rural areas, where the corresponding proportions of cancer deaths according to sources were 18·3%, 4·3%, 20·5% and 56·9% respectively. (This finding may be partly due to defects in the organization of registration of causes of death, *e.g.* registrars were content with information from relatives of the deceased and made no effort to search for evidence of causes of death from the medical facilities. A recent survey showed that in the rural areas of Shanghai only a small proportion of cancer patients (less than 3%) did not seek medical attention before dying).

Several factors which may influence objective evaluation of time trends (*e.g.* change of diagnostic criteria, improvement in diagnostic techniques, change of quality of registration and classification of causes of deaths) should be considered. First, no notable change or improvement of diagnostic methods for cancer in ordinary medical practice occurred during the period in question, except the introduction of cytological examination of sputum for lung cancer and the AFP test for primary liver cancer, both commonly used in recent years. The widespread use of cytological examination and AFP test could help to find early cases, but are not likely to explain the sharp increase in mortality rates for lung and liver cancer.

Secondly, the quality of registration of cancer deaths was somewhat worse during the period 1966–70 (see below) owing to the difficult circumstances during that time.

Percentage of cancer deaths without specification of site (Rubric 199 of ICD-8) during different periods

| | 1963–65 | 1966–70 | 1971–75 | 1976–77 | Total |
|---|---|---|---|---|---|
| Male | 1·57 | 4·08 | 0·89 | 0·06 | 1·78 |
| Female | 2·13 | 6·26 | 1·68 | 0·07 | 2·84 |

A high proportion of cancer deaths without specification of site in this period might artificially lower cancer mortality rates for some sites (but obviously not to a large extent) and, despite this temporary fall in standards, age-standardized mortality rates for sites such as lung, liver and pancreas for both sexes, or colon–rectum for females, were still higher in 1966–70 than those in 1963–65.

Thirdly, use of the 8th edition of The International Classification of Diseases for the coding of data for the whole, and the combination of some rubrics where assignation to one or another was doubtful, are helpful in avoiding problems in comparability between different periods.

During the period in question a rapid increase in mortality for lung cancer was parallelled by a sharp increase in the rates for cancer of bladder and pancreas among males. Possibly smoking might be closely linked to this finding and occupational factors might also be involved. But a similar increase in mortality for lung cancer and cancer of the pancreas was seen also among females (though mortality for bladder cancer rose less) and the smoking habit in females is much less frequent than in males. It seems that a detailed survey of smoking habits among inhabitants and its contribution to cancer should be carried out.

There was a dramatic decrease in the mortality rate for cancer of the uterus during the 15-year period. Comparing age-standardized rates between the 2 periods 1963–65 and 1971–75, it is found that the rate for malignant neoplasms of the cervix uteri (rubric 180 of ICD-8, A55) fell from 14·79 to 6·52 (the latter being 44% of the former, while the rate for chorionepithelioma and other malignant neoplasmas of the uterus (Rubrics 181–182, A56) fell from 5·12 to 3·49; thus the notable decrease in mortality rate for cancer of the uterus (180–182) stemmed mainly from the decrease in cancer of the cervix. This may be related to the large-scale mass screening for cancer of the cervix in the Shanghai urban area, which started in 1958 and continued thereafter. However, more information should be collected and analysed before making a firm conclusion.

Bearing in mind the weaknesses of the data presented in this report, cancer mortality experience in Shanghai may be summarized as follows:

1. The common sites of cancer in deaths from cancer among males in the Shanghai urban area were stomach, lung, liver, oesophagus and colon–rectum. Among females they were stomach, lung, uterus, liver, oesophagus, colon–rectum and breast.

2. During the period in question the crude cancer mortality rate in males increased by 109% and in females by 65%, but a large part of the increase can be explained by the change of age structure of the Shanghai population.

3. A rapid increase in the cancer mortality rate was seen for lung, colon–rectum, and pancreas in both sexes and for bladder in males. A notable decrease in mortality rate for cancer of the uterus occurred.

4. Overall cancer mortality rate was higher in the urban area. Higher rates were seen in the urban area for lung, oesophagus, and pancreas in both sexes, of the bladder in males, and of the uterus and breast in females, but the rate for liver cancer seemed to be higher in the rural areas. Virtually the same rates for large-bowel cancer and leukaemia in urban and rural areas suggest that, despite differences in sources of information, coverage may be comparable.

5. There were large differences between the mortality rates for cancer of the nasopharynx, stomach and oesophagus in Shanghai and the Singapore and Hong Kong populations. It would be very interesting to compare rates for migrants from Shanghai living in these cities.

The authors thank Dr C. S. Muir, International Agency for Research on Cancer (IARC), Lyon, France, for his valuable comments; the Unit of Research Training and Liaison of IARC for their help in preparing the graphs; Vital Statistics Section of Shanghai for collection of data on cancer deaths; and Mrs Zhou Shu Zhen, Miss Wang Xue Li, Mr Zhang Xing Fu and Mr Jiang Yi Lin, Department of Epidemiology of Shanghai Cancer Institute for their patient participation in processing the data and carrying out calculations.

### REFERENCES

SEGI, M. *et al.* (1978) *Age-adjusted death rates for cancer for selected sites in 52 countries in 1973.* Dept. of Public Health, Tohoku University School of Medicine, Sendai, Japan.

SHANGHAI MUNICIPAL BUREAU OF PUBLIC HEALTH (1962) *Regulation concerning notification of cancer cases and deaths.*

WATERHOUSE, J. A. H., MUIR, C. S., CORREA, P. & POWELL, J. (Eds) (1976) *Cancer Incidence in Five Continents, Vol. III.* Lyon: IARC Sci. Publ. **15**, 456.

WHO (1967) *International Classification of Diseases (1965 Revision).* Geneva: WHO.